# Modeling Diffusive Mixing in Antisolvent Crystallization

**DOI:** 10.1021/acs.cgd.1c01269

**Published:** 2022-03-14

**Authors:** Russell Miller, Jan Sefcik, Leo Lue

**Affiliations:** † Department of Chemical and Process Engineering, University of Strathclyde, James Weir Building, 75 Montrose Street, Glasgow G1 1XJ, U.K.; ‡ EPSRC Continuous Manufacturing & Advanced Crystallisation (CMAC) Future Manufacturing Research Hub, University of Strathclyde, Glasgow G1 1RD, U.K.

## Abstract

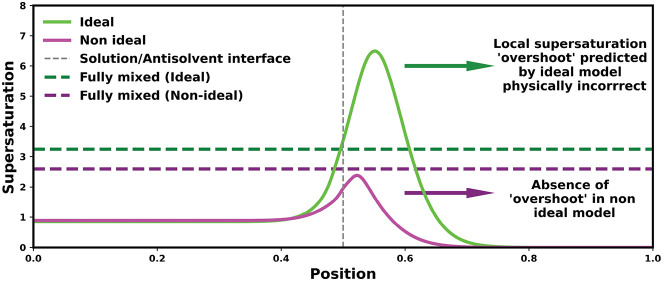

Diffusion controls local concentration profiles at interfaces between segregated fluid elements during mixing processes. This is important for antisolvent crystallization, where it is intuitively argued that local concentration profiles at interfaces between solution and antisolvent fluid elements can result in significant supersaturation overshoots over and above that at the final mixture composition, leading to poorly controlled nucleation. Previous work on modeling diffusive mixing in antisolvent crystallization has relied on Fickian diffusion, where concentration gradients are the driving force for diffusion. This predicts large overshoots in the supersaturation at interfaces between solution and antisolvent, as is often intuitively expected. However, chemical potential gradients provide a more physically realistic driving force for diffusion, and in highly nonideal solutions, such as those in antisolvent crystallization, this leads to nonintuitive behavior. In particular, as solute diffusion toward antisolvent is severely hindered, it can diffuse against its concentration gradient away from antisolvent. We apply thermodynamically consistent diffusion model based on the multicomponent Maxwell–Stefan formulation to examine diffusive mixing in a nonideal antisolvent crystallization system. Large supersaturation overshoots above that at the final mixture composition are not found when a thermodynamically consistent approach is used, demonstrating that these overshoots are modeling artifacts and are not expected to be present in physical systems. In addition, for certain conditions, localized liquid–liquid spinodal demixing is predicted to occur during the diffusive mixing process, even when the final mixture composition is outside the liquid–liquid phase separation region. Intermittent spinodal demixing driven by diffusive mixing may provide a novel explanation for differences of nucleation behaviors among various antisolvents.

## Abstract 2

Diffusion controls concentration profiles at interfaces between segregated fluid elements during mixing. In antisolvent crystallization, it is intuitively argued that local concentration profiles at solution/antisolvent interfaces can result in significant supersaturation overshoots leading to poorly controlled nucleation. These overshoots are not found when using a thermodynamically consistent approach, demonstrating that they are modeling artifacts and not expected in physical systems.

## 1 Introduction

Mass transfer phenomena play an important role in many chemical and physical processes, such as crystallization, and, therefore, it is essential to develop a better fundamental understanding of mass transfer effects in order to design more efficient crystallization processes. This Article focuses on the molecular diffusion aspect of mass transfer and on diffusive mixing in the context of antisolvent crystallization. This is a commonly employed crystallization process, in which the solute is crystallized from solution via the addition of a secondary solvent with poor solute solubility (antisolvent). The solubility in the resulting mixture is significantly lowered and crystallization of the solute is induced. Antisolvent crystallization offers flexibility in terms of achieving the desired supersaturation profiles through addition of a selected antisolvent to the solution. Multiple techniques are available to perform antisolvent addition allowing for further control over the mixing process. These include continuous static mixers or the injection of the antisolvent into a vessel containing the solution or vice versa.^1−3^

The development of effective crystallization processes requires careful thought of process parameters. For antisolvent processes these include initial solute concentration in the solution, antisolvent composition, antisolvent addition rate and mixing regime. To assist in the selection of these parameters, a modeling approach can be taken to provide insight into effects of these parameters on crystallization outcomes. Models can be developed to simulate the mixing process, and mixing models can be further combined with population balance models to develop integrated process models of antisolvent crystallization processes.^4^

Thermodynamically, the driving force for crystallization is the chemical potential difference between the solid phase and the solution. In crystallization, the driving force is generally expressed as supersaturation. Crystal nucleation and growth rates are sensitive functions of supersaturation, and therefore, the resulting crystal properties, such as solid form, crystal size distribution, and shape, are strongly influenced by supersaturation.^5^ During the mixing process during antisolvent addition, the composition of the system is not uniform and regions of high supersaturation are intuitively expected to exist, where the local supersaturation can exceed the final supersaturation value corresponding to the fully mixed solution. Nucleation would be more likely to occur in these localized regions of high supersaturations and different solvent compositions than under prevailing conditions in the fully mixed solution. Furthermore, the crystals produced may have solid forms differing from those expected. Local composition profiles are controlled via the mixing process, and therefore, an effective control of mixing is required for the design and operation of efficient antisolvent crystallization processes.^2^

Mixing can occur via two main mechanisms: diffusive and advective. Diffusion can be seen as mixing at a molecular level and occurs via random thermal motion, while advection is mixing at a bulk scale. The Peclet number is a dimensionless number that characterizes the ratio of convective to diffusive mixing and is expressed through *Pe* = *Lu*/*D*, where *L* is a characteristic length, *u* is a local fluid velocity, and *D* is diffusion coefficient. If the Peclet number is much less than unity, then mass transfer is considered diffusion dominated. Recently, applications of microfluidic crystallization have seen increased interest because of the high level of control provided.^6^ With the Peclet number significantly smaller than one in typical microfluidic setups, mixing can occur under conditions of free interfacial diffusion, resulting in strict control over supersaturation profiles and, hence, crystal properties. For Peclet number significantly greater than one, mixing is governed by two sequential processes, fluid bulk motion and molecular diffusion.^7^ Fluid motion acts to rearrange the spatial location of fluid elements, with the concentration of each species remaining unchanged within the element. Exchange of species between neighboring elements occurs via molecular diffusion and at sufficiently small length scales, mixing becomes dominated by diffusion. Therefore, diffusive mixing controls local supersaturation profiles under wide range of mixing conditions.

A variety of modeling approaches to simulate diffusive mixing have been used within the literature, however investigation of diffusive mixing applied within the antisolvent crystallization has been very limited. In a study on applications of microfluidics to polymorphic screening during antisolvent crystallization,^8^ the diffusive mixing during the antisolvent crystallization process was modeled using a Fickian approach assuming thermodynamically ideal solutions. Insights obtained from this modeling were qualitatively compared to experimental observations on crystallization in microfluidic wells composed of two adjacent chambers, one with filled with antisolvent and the other with an indomethacine solution, where diffusive mixing occurred at the interface between the two chambers. The wells allowed for various ratios of antisolvent to solution to be used, and the effect on nucleation outcomes were investigated. It was concluded that the development of the spatiotemporal supersaturation profiles plays a key role in the formation of crystals, in terms of both properties and location of crystallization, although relationships between calculated supersaturation profiles and observed nucleation outcomes were not straightforward.

In a later study,^9^ a continuous microfluidic platform was used in the polymorphic screening of glycine. Glycine was crystallized through antisolvent crystallization in which diffusive mixing takes place at laminar flow interfaces. The mixing process was modeled by solving the steady-state Navier–Stokes equation to obtain the velocity profile of the streams, which was then inserted into the steady-state convective-diffusive equation, again using a Fickian approach assuming thermodynamically ideal solutions. This approached was used to calculate the concentration and supersaturation profiles of the microfluidic channel. The model prediction showed a wide variation in the local supersaturation throughout the channel, including significant overshoots above supersaturation of the final mixed solution. This would, then, imply the possible formation of several different glycine polymorphs, where both α- and β-glycine were observed in corresponding experiments.

In the previous studies, diffusive mixing is described within a Fickian framework, where concentration gradients are used as the driving force, with intuitions developed based on this. However, the driving force for diffusion are more physically rooted in chemical potential gradients, and nonideal thermodynamic solution behavior needs to be taken into account.^10^ For example, the solute would not be expected to diffuse into the antisolvent, as this would be against the thermodynamic driving force, while it would be allowed in the Fickian diffusion framework.

In this work, we will model multicomponent diffusion within the Maxwell–Stefan framework, where diffusion is driven through chemical potential gradients.^10^ To study diffusive mixing in antisolvent crystallization, we consider ternary solutions of glycine (0), water (1), and ethanol (2). Glycine is the solute, water is the solvent, and ethanol or a mixture of ethanol/water is used as the antisolvent. This specific antisolvent crystallization system was chosen because it is well studied experimentally in the literature and thermodynamic data are readily available. Furthermore, this system is representative of typical antisolvent systems, placing this work in the wider context of antisolvent particle formation processes. Composition and supersaturation profiles will be calculated across diffusion interfaces and ternary phase diagrams. While crystallization as such is not modeled in this work, relationships between process parameters and crystallization outcomes can be inferred from the corresponding supersaturation profiles. A comparison will be made between diffusive mixing in ideal and nonideal solutions, revealing qualitative differences in their respective behaviors and challenging previous intuitions based on a Fickian framework. This work provides novel insights into the role of chemical potential gradients in diffusive mixing during antisolvent crystallization, which will assist in the development and design of more efficient crystallization processes.

The remainder of this Article is organized as follows: In the following section, we present the free energy model used to describe the solution thermodynamics of the system and its parametrization. In section 3, the details of the Maxwell–Stefan diffusion model are presented, and the parameters of the model, such as the mutual diffusion coefficients, are determined. The details of the diffusion simulations are provided in section 4. Section 5 then compares the results between ideal solutions, where diffusion is driven by concentration gradients, and nonideal solutions, where diffusion is driven by chemical potential gradients. Finally, the main findings of the work are summarized in section 6.

## 2 Thermodynamics of Glycine/Water/Ethanol/Mixtures

### 2.1 Thermodynamic Model

The extended Scatchard–Hildebrand model,^11^ which includes the effect of size asymmetry in the entropy of mixing in the standard regular solution model,^12^ is used to describe the thermodynamics of the ternary water/ethanol/glycine mixtures. The molar Gibbs free energy *G* is given by
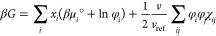
1where μ_*i*_° is the chemical potential of pure component *i* in the liquid state at the system temperature, β = 1/(*RT*), *R* is the gas constant, *T* is the absolute temperature, *v*_ref_ is the reference volume (which we will take to be the solvent volume), *v* = ∑_*i*=1_^*N*^*x*_*i*_*v*_*i*_ is the molar volume of the solution, *x*_*i*_ and *v*_*i*_ are the mole fractions and effective component molar volume, respectively, φ_*i*_ = *x*_*i*_*v*_*i*_/*v* is the volume fraction of species *i*, and χ_*ij*_ is a binary interaction parameter between species *i* and *j*. The binary interaction parameters are symmetric (i.e., χ_*ij*_ = χ_*ji*_), and χ_*ii*_ = 0. Physically, they represent the incompatibility between species; the larger the value of χ_*ij*_, the greater the tendency for components *i* and *j* to demix. The effective molar volumes *v*_*i*_ and the binary interaction χ_*ij*_ are taken as adjustable parameters of the thermodynamic model and are obtained by fitting available experimental data. The ideal solution model corresponds to the situation where all the species volumes *v*_*i*_ have the same value and the binary interactions χ_*ij*_ are equal to zero.

The chemical potential μ_*i*_ of component *i* can be written as
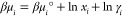
2where γ_*i*_ is the activity coefficient of *i*. The sum of the first two terms is the chemical potential of component *i* in an ideal solution, while the final term represents the contribution of solution nonideality. In an ideal solution, γ_*i*_ = 1 for all species. In the extended Scatchard–Hildebrand model, the corresponding expression for the activity coefficient of component *i* in a multicomponent mixture:
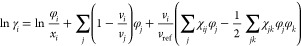
3

Along the solubility curve, the chemical potential of glycine in the liquid mixture is the same as that for pure crystalline glycine, which leads to the relation
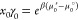
4where μ_0_^*s*^ is the chemical potential of pure solid glycine. The right side of eq 4 is independent of solution composition, and we define a solubility constant as β(μ_0_^*s*^ – μ_0_^°^). This constant and the glycine/ethanol binary interaction parameter (i.e., χ_02_) were fitted to solubility data for glycine in water/ethanol solvent mixtures. The solubility data used were a combination of literature values^2^ and solubility measured in this work.

The left side of eq 4 is the activity of glycine. When it becomes larger than the right side of eq 4, the solution is supersaturated, and glycine will tend to crystallize from solution. We define the supersaturation ratio as *S* = *x*_0_γ_0_*e*^–β(μ_0_^*s*^–μ_0_^°^)^. When *S* < 1, the solution is under saturated, when *S* = 1, the solution is saturated, and when *S* > 1, the solution is supersaturated.

### 2.2 Model Parameters

**Figure 1:**
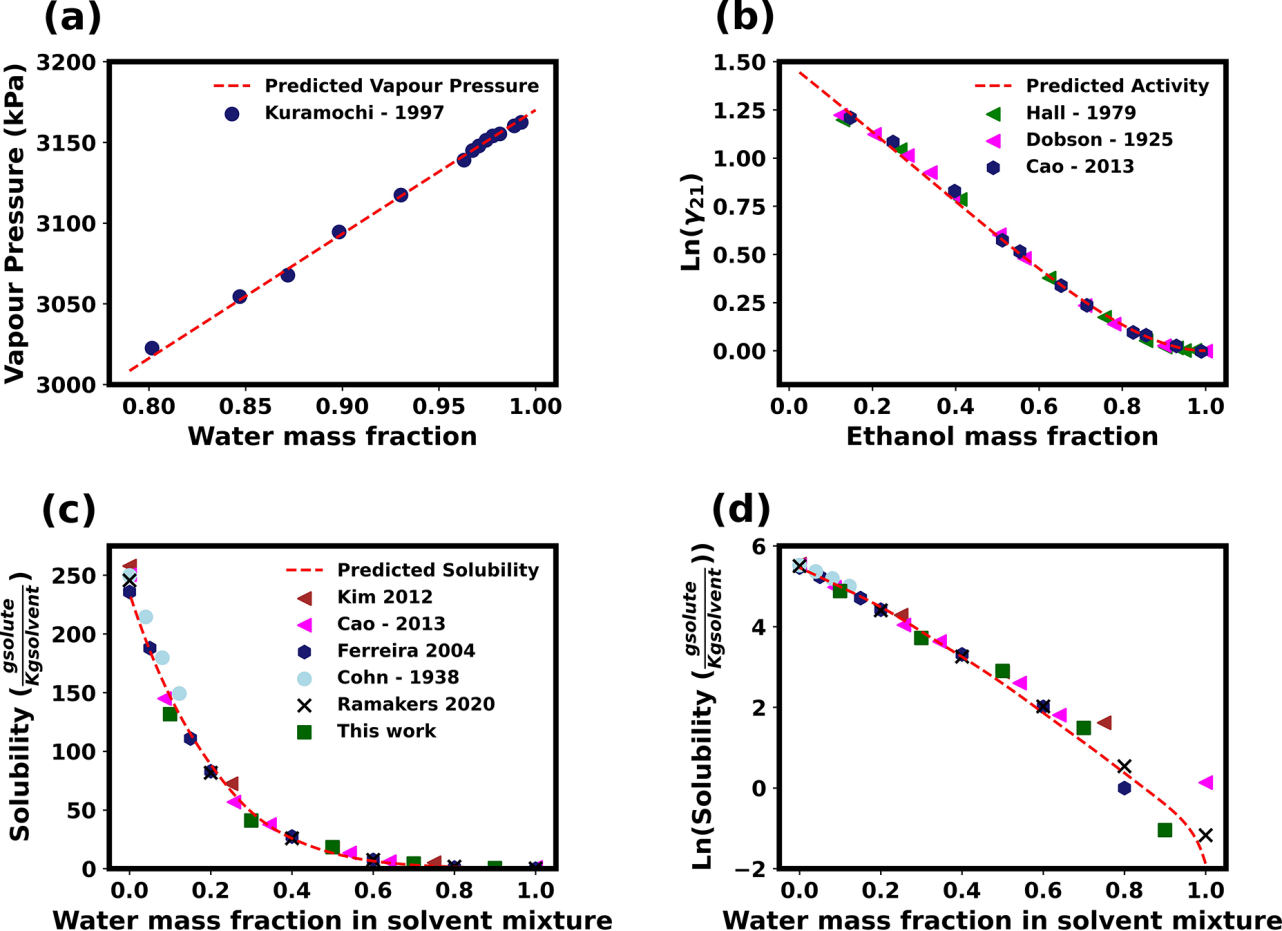
(a) Comparison of the predicted vapor pressure with measurements from the literature for water/glycine binary system. (b) Predicted activity coefficient of ethanol in water/ethanol mixtures compared to values derived from experimental VLE data taken from literature. (c) Solubility of glycine in water/ethanol mixtures as predicted by the thermodynamic model at 298 K. Green squares show the solubility measured in this work through gravimetric analysis. (d) Solubility shown on a logarithmic scale.

For a ternary solution, there are three binary interaction parameters. The glycine/water interaction parameter χ_01_ and glycine volume relative to water *v*_0_/*v*_1_ were fitted to vapor pressure measurements for glycine/water mixtures.^13^ Values for the binary interaction χ_12_ and ethanol volume with respect to water *v*_2_/*v*_1_ were obtained by fitting experimental VLE data for water/ethanol mixtures,^14−16^ across a wide composition range. The fits of the thermodynamic model are shown in Figure 1a and b. For both cases, the activity model fit the data well, and a reasonable estimate of the interaction parameters was obtained.

Measurements were performed using gravimetric analysis to obtain accurate solubility data for glycine in water–ethanol mixtures, which are required to parametrize the thermodynamic model used in this work. Glycine (≥99%) was sourced from Sigma-Aldrich, ethanol (≥99.8%) was supplied by VWR. Deionized water was used to prepare the aqueous glycine solution. Slurries of glycine in water/ethanol mixtures were added to 8 mL vials containing magnetic stirrer bars and the vials were placed on a submersible stirrer plate in a water bath set to 25 °C. The stirring speed was set to 700 rpm. At least 3 vials for each solvent composition were used, with more vials used for solvent mixtures containing low amounts of water, to ensure results were reproducible. The vials were left for 72 h to allow the slurry to reach equilibrium. After 72 h, a syringe was used to withdraw 2 mL of the clear mother liquid and inject into an empty preweighed vial. A syringe filter was used to ensure no undissolved glycine was transferred into the new vial. The new vials containing the clear mother liquor were placed in a vacuum oven to evaporate the solvent. Once all solvent had been evaporated, the mass of glycine was determined and the solution concentration was calculated from material balances.

The measured solubilities, together with data previously reported in the literature,^2,16−19^ are shown in Figure 1c. The solubility measured in this work (green squares) is in a good agreement with the literature values. Some variation of solubility was observed at low water mass fractions in the solvent mixture, as highlighted in Figure 1d. This is due to the challenges associated with measuring the extremely low glycine concentrations in mixtures with little water content. The glycine/ethanol interaction parameter χ_02_ and the solubility constant β(μ_0_^*s*^ – μ_0_^°^) were determined by fitting the solubility of glycine in water/ethanol solvent mixtures measured in this work. The red dotted line shows the predictions of the fitted thermodynamic model.

The values of the fitted parameters used in the thermodynamic model are summarized in Table 1. The ternary phase diagram for our system can be calculated from the free energy model.

**Table 1:** Values of the Parameters Used in the Thermodynamic Model

parameter		value
water/glycine binary interaction parameter	χ_01_	0.59
ethanol/glycine binary interaction parameter	χ_02_	2.075
water/ethanol binary interaction parameter	χ_12_	1.07
solubility constant	β(μ_0_^*s*^ – μ_0_^°^)	–2.2
glycine relative volume	*v*_0_/*v*_1_	3.58
ethanol relative volume	*v*_2_/*v*_1_	1.50

**Figure 2:**
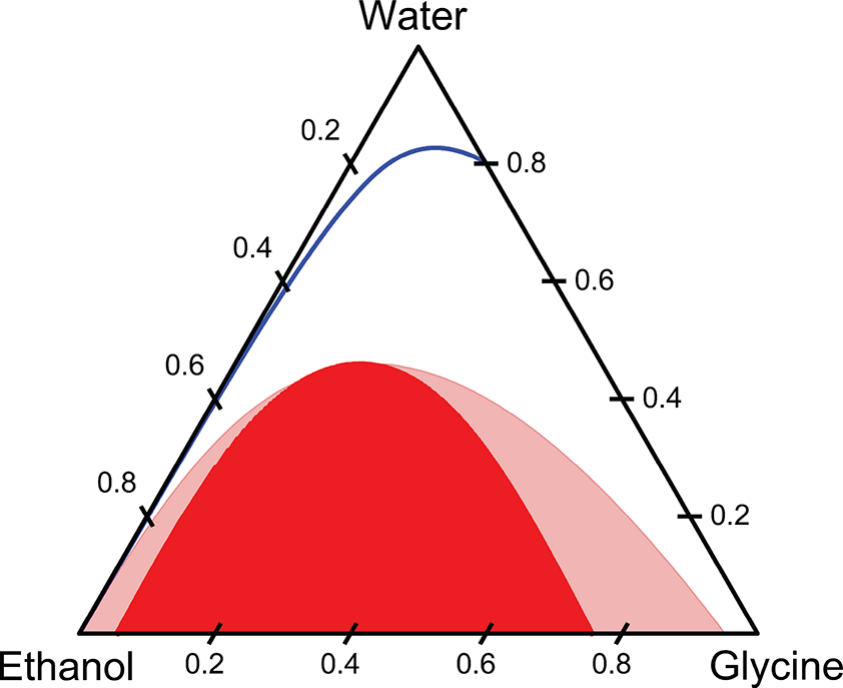
Ternary phase diagram for the glycine/water/ethanol system on a mass fraction basis. The calculated glycine solubility curve is shown with the blue line. The binodal region is shown as the light red shaded area, and the spinodal region is shown as the dark red shaded area on the ternary phase diagram.

The requirement for phase stability is that the eigenvalues of the thermodynamic factor (Γ, discussed in the multicomponent diffusion section) must be positive (i.e., det Γ > 0).^10^ This property was used to determine compositions where the solution is predicted to be unstable. The binodal curve is determined by equating the chemical potential of each species in both coexisting phases phases. The predicted phase diagram is shown in Figure 2 on a mass fraction basis. The glycine solubility curve is given by the blue line. There is a liquid–liquid phase coexistence region predicted by the thermodynamic model, which is shaded in red; the lightly shaded region is the binodal, where the solution is metastable, while the darkly shaded region is the spinodal, where the solution is unstable and will spontaneously split into two phases. Note that this entire two-phase region is metastable with respect to glycine crystallization.

The thermodynamic model (i.e., eq 1 with parameters given in Table 1) predicts a region of liquid–liquid phase separation, which is metastable with respect to the solid–liquid phase coexistence. For concentrations within the spinodal region indicated by bright red, the solution is unstable and will spontaneously separate into two liquid phases. Consequently, the model predicts that liquid–liquid phase separation (LLPS) can occur under certain conditions during the antisolvent crystallization processes, which may be experimentally observed as “oiling out” of the solution prior to crystallization.

## 3 Multicomponent Diffusion

The dynamics of the species in solution are governed by the conservation equation, which relates the local accumulation of a species to its local flux.
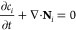
5where *c*_*i*_ is the local molar concentration of species *i*. The total species flux **N**_*i*_ can be separated into a convective and a diffusion contribution as
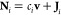
6where **v** is a fluid velocity and **J**_*i*_ is the diffusive flux. The division between the two types of fluxes is somewhat arbitrary and dependent on the choice of the definition of the fluid velocity, such as a center of mass velocity, molar velocity, or solvent velocity.^20^ While the choice of reference frame does not impact the physics of a system, some choices are more convenient than others depending on its particular boundary conditions. In this work, we deal with a sealed liquid system, where there is little volume change of mixing, so the molar volumes of each component can be assumed to be constant (and equal to its volume in the pure state). In this case, it is natural to use the fluid velocity based on the volume reference frame, which is defined by
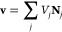
7where *V*_*j*_ is the volume occupied by species *j*.

The corresponding diffusive fluxes satisfy the relation:
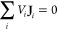
8Note that the component volumes *V*_*i*_ used in the definition of the volume reference frame are distinct from the volumes *v*_*i*_ used in the free energy model developed in section 2. In this work, *V*_*i*_ is taken to be the molar volume of the pure component *i* and represents the space occupied by a molecule; their values are summarized in Table 2. The volumes *v*_*i*_ are considered to be fitting parameters in the free energy model that are chosen to reproduce the thermodynamic properties of the system, such as the species activity coefficients.

**Table 2:** Parameters of the Diffusion Model

parameter	value	values investigated
glycine relative molar volume: *V*_0_/*V*_1_	3.58	
ethanol relative molar volume: *V*_2_/*V*_1_	3.23	
*Đ*_01_/10^–9^ m^2^ s^–1^	1	0.40, 1.00, and 1.25
*Đ*_02_/10^–9^ m^2^ s^–1^	1	0.40, 1.00, and 1.25
*Đ*_12_/10^–9^ m^2^ s^–1^	1	0.40, 1.00, and 1.25

In this work, the Maxwell–Stefan approach is used to describe the molecular diffusion in the system.^10,21^ Within this theoretical framework for an isothermal system, the diffusive fluxes of each molecular species **J**_*i*_ are driven by the gradients in the chemical potentials μ_*i*_ through the relations:
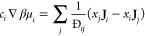
9where *c*_*i*_ is the concentration of species *i*, and *Đ*_*ij*_ is the mutual diffusion coefficient between species *i* and *j*. For a ternary mixture, this can be formally inverted to give explicit formulas for the diffusive fluxes in the volume reference frame^20^
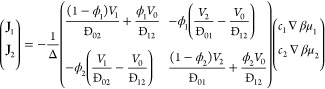
10where ϕ_*i*_ is the volume fraction of species *i* defined in terms of the volumes *V*_*i*_.

More commonly, Fick’s law is used to relate the diffusive flux to the composition gradient within a mixture. For a ternary component system, diffusive flux **J**_*i*_ of component *i* is given by^22^
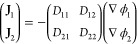
11where *D*_*ij*_ is the Fickian diffusion coefficient of component *i* in *j*.

The Maxwell–Stefan expression for the diffusive fluxes, given in eq 10, can be recast in the Fickian form for diffusion, providing a relation between the Fickian diffusion coefficients and the Maxwell–Stefan diffusion coefficients:
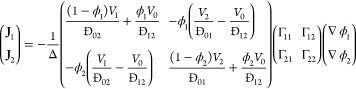
12where Γ_*ij*_ is known as the matrix of thermodynamic factors and is defined as
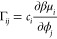
13and Δ is given by
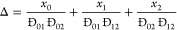
14The matrix Γ describes the impact of nonideal solution behavior on diffusion and can be calculated from a thermodynamic model, such as the one developed in section 2. For an ideal solution, Γ reduces to the identity matrix. In this work, the ideal solution is defined for the case χ_01_ = χ_02_ = χ_12_ = 0 and *v*_0_ = *v*_1_ = *v*_2_. This ignores the contribution due to the size differences of the molecules and their mutual interactions, and results in an activity coefficient of 1.

The Maxwell–Stefan approach has several advantages to the more commonly used Fickian description of diffusion. Unlike the Fickian diffusion coefficients, the Maxwell–Stefan coefficients *Đ*_*ij*_ are symmetric;^10^ therefore, for a ternary mixture, only three diffusion coefficients are required. In addition, the Fickian diffusion coefficients (*D*_*ij*_ in eq 11) are dependent on the reference frame; their values depend on the particular reference frame that is selected. Furthermore, these values are dependent on concentration, pressure, and temperature.^22^ For binary mixture, the simplicity of Fickian diffusion has seen it become the standard for interpreting experimental measurements, and binary diffusion coefficients are typically reported in terms of Fickian diffusivity. For multicomponent mixtures, the situation becomes more complicated with the diffusivity being expressed by a nonsymmetric (*n* – 1) × (*n* – 1) matrix for an *n*-component mixture.

**Figure 3:**
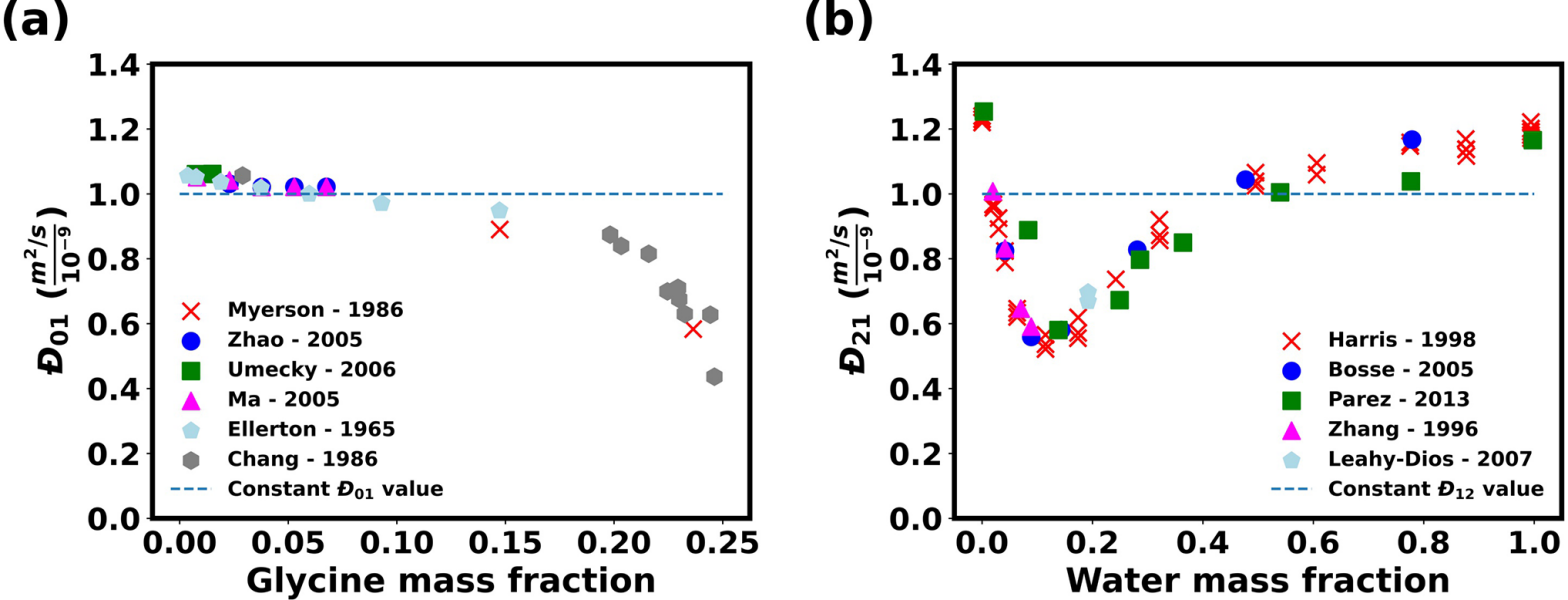
Maxwell–Stefan diffusion coefficients for (a) water/glycine *Đ*_01_ and (b) water/ethanol *Đ*_12_. The gray dashed line indicates the representative value for the diffusion coefficient used in this work.

Estimates for the Maxwell–Stefan diffusion coefficients were obtained from Fickian diffusion coefficients reported in the literature for binary glycine–water^23−28^ and ethanol–water^29−33^ mixtures, through the use of eqs 11–13, combined with the thermodynamic model developed in section 2. The results are shown in Figure 3.

For simplicity in the calculations in this work, the Maxwell–Stefan diffusion coefficients were assumed to be independent of composition, and a nominal value of 10^–9^ m^2^ s^–1^ was selected for each pair of binary diffusion coefficients. The diffusion coefficient for ethanol/glycine was unknown, and it was chosen to be of a the same magnitude. To explore the range of plausible behaviors, a parametric study was performed for the values of diffusion coefficients used. The value ranges selected were based on the maximum and minimum values experienced over the compositions ranges for water/ethanol and water/glycine. The range of values for the ethanol/glycine were selected to be of similar magnitudes. This analysis was carried out for both ideal and nonideal diffusion. Table 2 summarizes the values of diffusion coefficients used in the model.

## 4 Simulation Details

We examined systems confined within a closed, rigid channel of width 1 mm. The left side is initially filled with an aqueous glycine solution, and the right side is initially filled with the antisolvent or an antisolvent/solvent mixture. An example of the initial volume fraction profile is given in the Supporting Information. The solution and antisolvent were allowed to freely diffuse into each other, with no convective mixing taking place. The system was assumed to be one-dimensional.

The temperature was assumed to be constant at 298 K, and heat of mixing was neglected. The components chosen in this work form a nonideal mixture; however, to simplify the model, the volume change upon mixing was not considered. Nucleation is not considered in this model, although the calculated supersaturation profiles will provide qualitative insight into the propensity for crystal formation. Diffusive mixing is first assumed to be ideal, before accounting for nonidealities via the inclusion of the thermodynamic model.

Diffusive mixing in antisolvent crystallization was modeled by using eq 5. In this work, the system is approximately incompressible, and the convective flux in the volume frame is nearly stationary (i.e., **v** ≈ 0). In this case, it is natural to use the diffusive fluxes in the volume frame. The species conservation equation then becomes
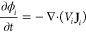
This formulation ensures that ∑_*i*_ ϕ_*i*_(**r**, *t*) = 1. Neumann boundary conditions were imposed on the channel walls: **n̂**·∇ϕ_*i*_ = 0 (i.e., no flux at channel walls). The parameters of the diffusion model are summarized in Table 2.

These diffusion equations were numerically solved using the finite volume solver FiPy v3.4^34^ on a regular one-dimensional domain with 1024 mesh points and a time step of 0.1 s. The number of mesh points and size of the time step were varied to ensure that the solution was accurate and independent of their particular choice.

To study the effect of key process parameters on supersaturation profiles, the initial volume fraction profile across the channel was varied. The parameters investigated were the initial antisolvent composition, ratio of antisolvent to solution within the channel and the initial glycine concentration in the aqueous glycine solution. Table 3 summarizes the parameters and the ranges of values used. The antisolvent was a mixture of water and ethanol. Supersaturation profiles were calculated from the volume fraction profiles obtained from the model using the thermodynamic model described in section 2. The results from this are shown in the Supporting Information. In this work, the supersaturation of glycine is defined as the ratio of local activity in the solution and its activity in a saturated solution with the same local solvent composition (i.e., *S* = *x*_0_γ_0_*e*^–β(μ_0_^*s*^–μ_0_^°^)^). The activity coefficient of glycine in the solvent mixture is calculated via eq 3.

**Table 3:** Key Process Parameters

process parameter	range
initial antisolvent composition	pure ethanol–50 vol % ethanol
antisolvent: solution ratio in channel	9:1–3:7
initial glycine volume fraction in solution	0.08–0.23

Diffusion in nonideal liquid mixtures was simulated and compared both qualitatively and quantitatively to the ideal case. With nucleation most likely to occur within the region of peak supersaturation, the compositional trajectories of the peaks were plotted on a phase diagram for both ideal and nonideal diffusion. This highlights the qualitative differences in the mixing process, and in particular, the differences in local composition of the peak supersaturation. The phase diagram indicates the spinodal region, and based on the trajectory of the peak supersaturation, spinodal decomposition can be predicted to occur. In terms of crystallization, nucleation could occur in the oiled out phase, thus significantly effecting local composition and, therefore, nucleation outcomes.

## 5 Results and Discussion

### 5.1 Effect of Nonideality on Diffusive Mixing

In previous work on modeling of diffusive mixing in antisolvent crystallization processes, the solutions are usually assumed to be ideal, and the diffusion of a species is taken to be driven by its local concentration gradient. However, antisolvent crystallization systems are necessarily highly nonideal, and the assumption of ideality fails to properly represent the key feature of the system—the tendency of the solute to avoid mixing with the antisolvent. Chemical potential (or equivalently activity) gradients, rather than the composition gradients, fundamentally drive species diffusion,^10,21^ and so nonideal mixing is expected to play a significant role in the dynamics of the system.

**Figure 4:**
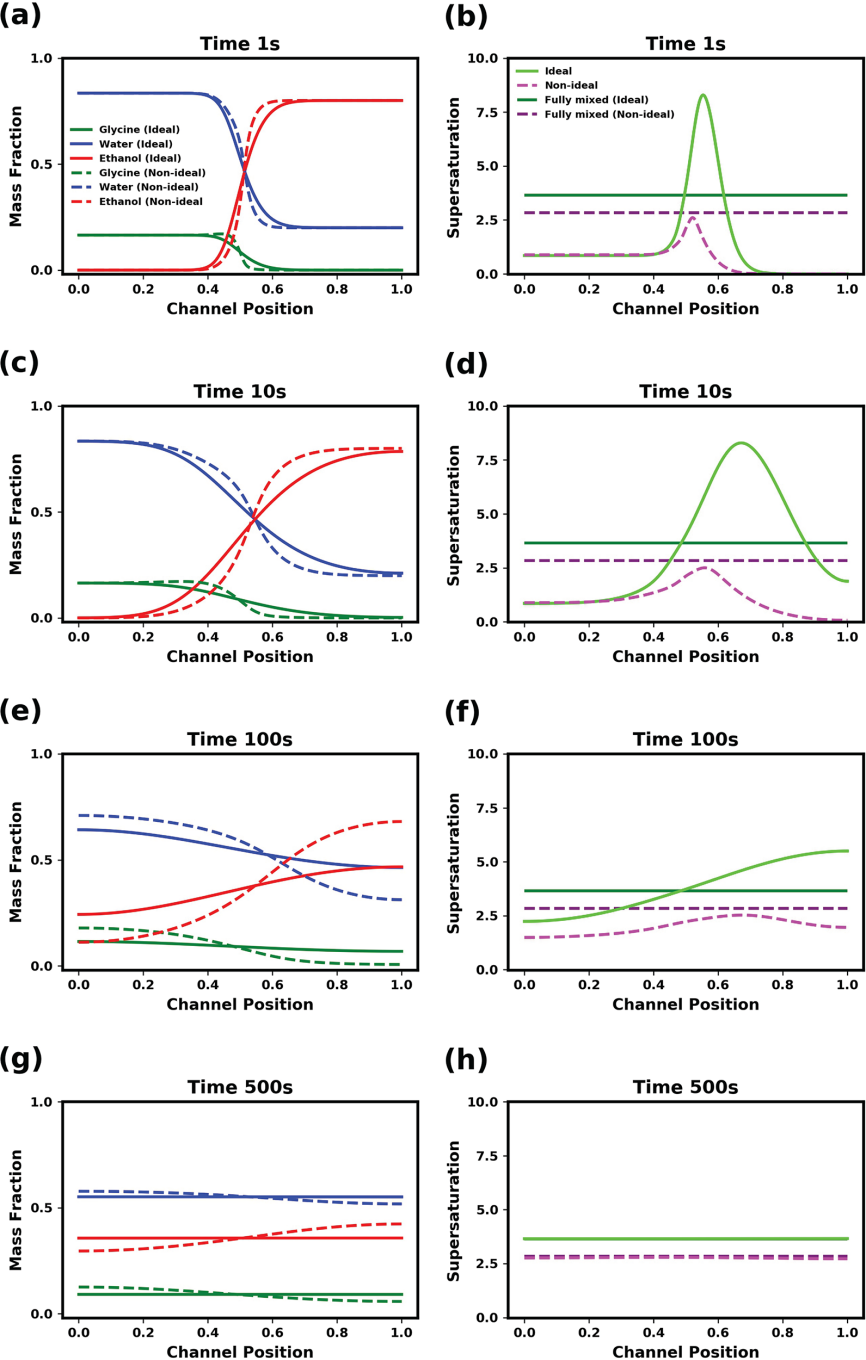
Comparison of diffusion mixing in ideal and nonideal solutions. The mass fraction and supersaturation profiles are shown at various times for ideal (solid lines) and nonideal (dashed lines) diffusion. The initial supersaturation in the solvent is 0.85 for the ideal model and 0.89 for the nonideal model, with nominal composition being the same for both models. The initial antisolvent composition was 80 wt % ethanol and 20 wt % water. The mutual diffusion coefficients were all 10^–9^ m^2^ s^–1^. The channel position is in reference to simulated channel described in the methods section. The initial solution/antisolvent interface is at 0.5 mm.

To examine the influence of nonideal mixing on diffusion, the time evolution of the concentration profiles are compared in Figure 4a, c, e, and g. The solid lines correspond to the ideal systems, while the dashed lines correspond to the nonideal systems. The corresponding supersaturation profiles are shown in Figure 4b, d, f, and h.

Differences in the diffusion between the ideal and nonideal mixtures are most apparent in the concentration profile of glycine. In the ideal mixture, the driving force is the composition gradient (which is identical with the activity in this case), and glycine diffuses from a region of relatively high concentration in the solvent to a region of relatively low concentration in the antisolvent. Because of the low solubility of glycine in the antisolvent mixture, a large peak in supersaturation is rapidly generated at the interface between the glycine solution and the antisolvent. At 1 s (see Figure 4a and b), a large overshoot in supersaturation is observed (i.e., a peak above the value of the supersaturation in the final fully mixed system). As mixing proceeds, the peak of supersaturation curve moves toward the antisolvent side of the channel. This supersaturation peak slowly flattens and eventually vanishes as water diffuses into the antisolvent, increasing the local solubility of glycine. On the solution side of the channel, interdiffusion of the solution and the ethanol leads to a reduction of solubility. As a consequence, the supersaturation of glycine gradually increases to the final, fully mixed value. By 500 s, the supersaturation becomes uniform across the channel as the composition gradients within the system relax.

In the nonideal mixture, activity gradients drive the diffusion, and glycine will diffuse away from the antisolvent, which is “uphill” with respect to its composition gradient. As water interdiffuses with the ethanol, glycine is dragged with the water into the antisolvent. This generates a peak in the supersaturation profile at the solution/antisolvent interface, however, due to the tendency of the glycine to diffuse away from ethanol, this does not lead to an overshoot, where supersaturation exceeds the final, fully mixed value (denoted by the dashed dark purple line) because of the relatively low concentration of glycine in the antisolvent. This is one obvious difference from the ideal system.

Another difference between the two is that the concentration profiles evolve more slowly in the nonideal system. Considering Figure 4g and h, while the supersaturation profile within the channel are nearly uniform, approaching that of the fully mixed system, composition gradients are still present in the nonideal system. This emphasizes that the physical system acts primarily to smooth out any gradients in chemical potential as opposed to compositional gradients. As the activity gradient flattens, the driving force for diffusion decreases, which is reflected in the comparatively longer times for the composition profiles to become uniform in the nonideal systems, as compared to faster relaxation in ideal systems.

### 5.2 Influence of Relative Diffusivities

**Figure 5:**
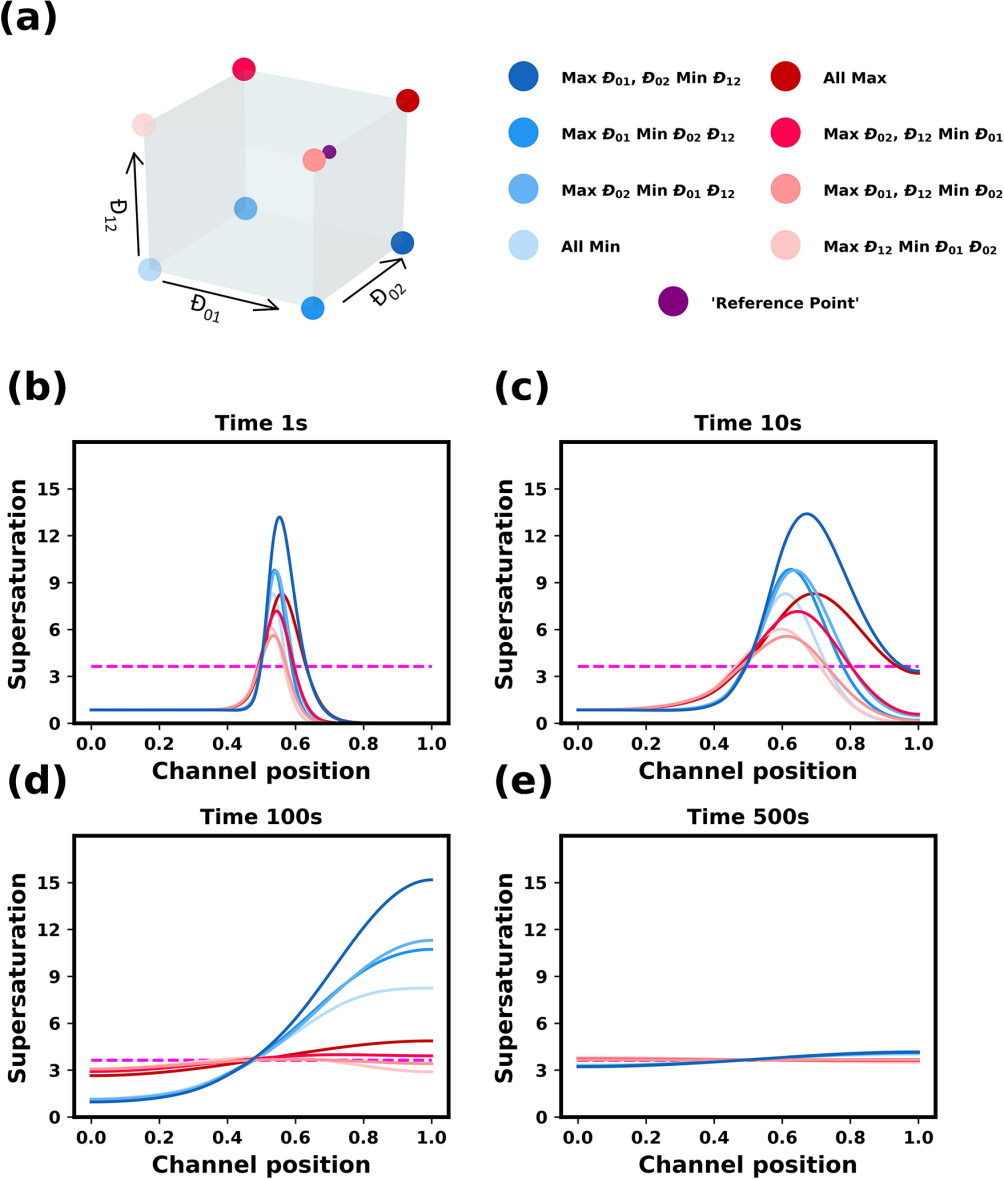
Influence of the mutual diffusion coefficients on the dynamics of ideal solutions. (a) The corners of the cube represent the 8 sets of diffusion coefficients used. The purple point indicates the “reference point” values used in Figure 4. (b)–(e) Evolution of supersaturation profiles at 1, 10, 100, and 500 s. The initial supersaturation in the solvent is 0.85. The initial antisolvent composition was 80 wt % ethanol and 20 wt % water. The horizontal dashed magenta line shows the value of the supersaturation value of the fully mixed conditions. The channel position is in reference to simulated channel described in methods section. The initial solution/antisolvent interface is at 0.5 mm.

The above analysis assumed that all the mutual diffusion coefficients were equal to 10^–9^ m^2^ s^–1^. In this section, we examine the influence of the relative values of the three mutual diffusion coefficients on the behavior of the system. Each mutual diffusion coefficient was chosen to have one of two values: a low value of 0.4 × 10^–9^ m^2^ s^–1^ or a high value of 1.25 × 10^–9^ m^2^ s^–1^. These values encompass the range of diffusion coefficients experimentally observed across relevant composition ranges (see Figure 3). Simulations were performed for each of the eight combinations of values of the mutual diffusion coefficients, which are depicted graphically in Figure 5a. The sensitivity of predicted supersaturation profiles with reference to the relative values of mutual diffusion coefficients can be determined by considering the temporal variation of the supersaturation profiles for each combination of values used.

We begin our investigation with ideal solutions. Figure 5 shows supersaturation profiles at various times for ideal solutions with different sets of mutual diffusion coefficients. For all systems, the supersaturation profiles form a peak at the solvent/antisolvent interface, and this peak broadens and moves deeper into the antisolvent, eventually meeting the right wall of the channel and then gradually becoming uniform. However, the precise evolution of the supersaturation profiles is sensitive to the relative values of binary coefficients used in the model. Moments after the onset of mixing, the systems appear to divide into two groups: those with higher supersaturation overshoots where the water/ethanol mutual diffusion coefficient *Đ*_12_ is small, and those with lower supersaturation overshoot where the water/ethanol diffusivity is large.

The systems with the higher supersaturations, which have a blue shade in Figure 5, correspond to conditions in which the minimum value of the glycine/ethanol diffusion coefficients is used (i.e., 0.4 × 10^–9^ m^2^ s^–1^). This is expected, as glycine will diffuse into the antisolvent mixture at a faster rate than water. This causes the antisolvent mixture to have a relatively higher fraction of ethanol, which implies that the solubility of glycine in the antisolvent will remain low, resulting in a large overshoot in the local supersaturation. For these systems, the height of the peak continues to grow as it moves into the antisolvent mixture. They also relax more slowly to the uniform profile, but this can be directly attributed to the lower value of *Đ*_12_.

The systems with the lower supersaturations, which have a red shade in Figure 5, have a higher value of the water/ethanol mutual diffusion coefficient. Because the water and ethanol mix more quickly, glycine solubility in the antisolvent mixture increases more rapidly, which prevent the local supersaturation from becoming very large. For these systems, the height of the supersaturation peak decreases as it moves into antisolvent mixture. We also note that in situations where glycine diffuses more slowly, the glycine concentration in the antisolvent mixture increases gradually, allowing water to more time to mix with ethanol, which leads to lower supersaturations.

In summary, the quantitative behavior of the spatiotemporal evolution of the supersaturations profiles in ideal solutions appears to be controlled mainly by the magnitude of the water/ethanol mutual diffusion coefficient, with slower water/ethanol mixing leading to a larger overshoot of the local supersaturation.

**Figure 6:**
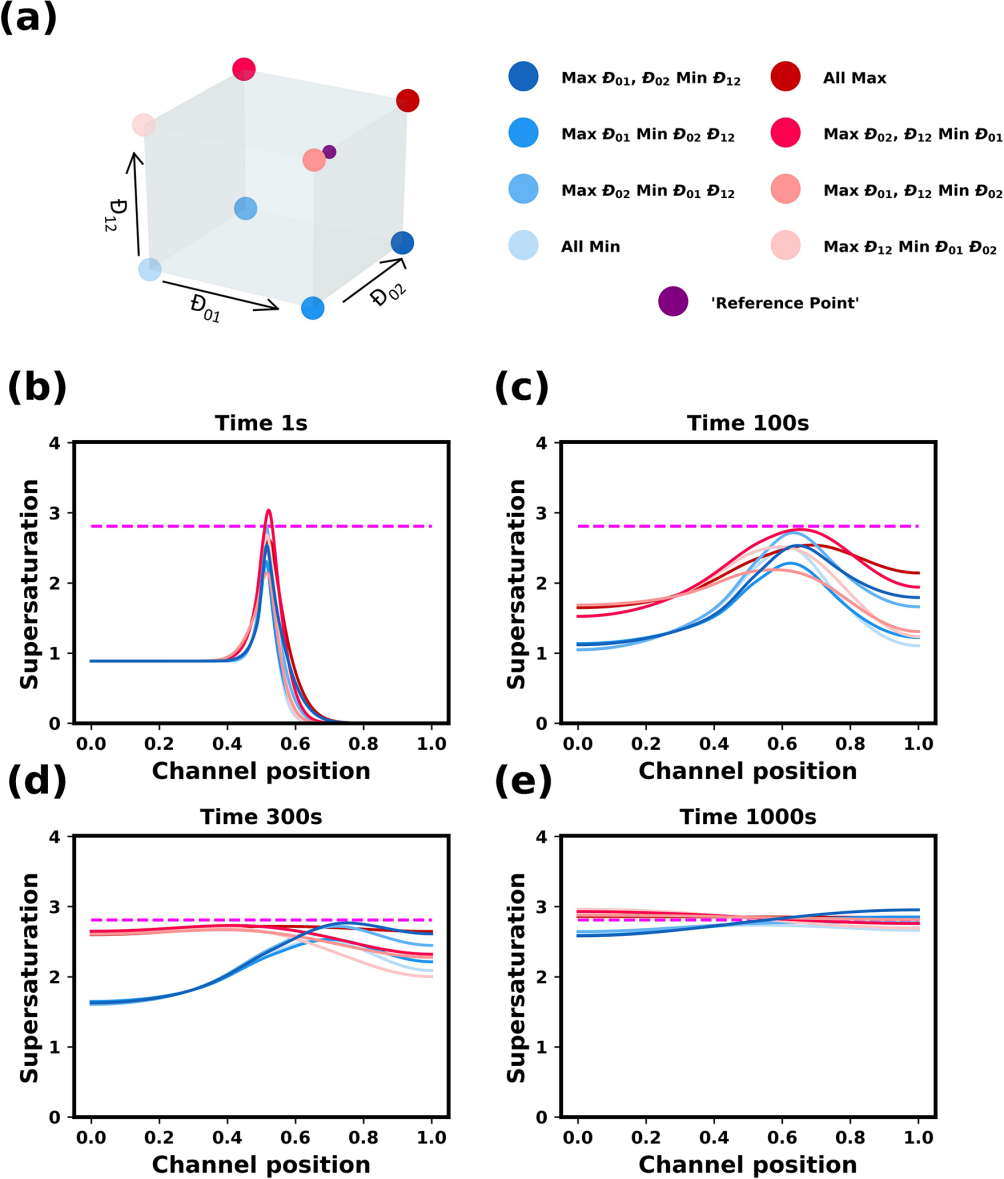
Influence of the mutual diffusion coefficients on the dynamics of nonideal solutions. (a) Cube plot with corners showing the 8 sets of diffusion coefficients used. The purple dot indicates the “reference point” values used in Figures 4. (b)–(e) Evolution of supersaturation profiles at times 1, 100, 300, and 1000 s. The initial supersaturation in the solvent is 0.89. The initial antisolvent composition was 80 wt % ethanol and 20 wt % water. The horizontal dashed magenta line shows the supersaturation value of the fully mixed conditions. The channel position is in reference to simulated channel described in methods section. The initial solution/antisolvent interface is at 0.5 mm.

Following the same approach as for ideal solutions, a parametric study of the diffusion coefficients in nonideal mixtures was carried out, and the evolution of the supersaturation profiles are shown in Figure 6. As before, a local peak in the supersaturation appears at the solvent/antisolvent interface. A key difference from the ideal case, as observed in the previous section, is that the local supersaturation does not, in general, overshoot the final, fully mixed value. Only for one set of diffusion coefficients does it slightly exceed the fully mixed value, however, then it quickly falls below the final supersaturation.

Similarly to the ideal case, two groups can be seen to form, driven by the difference in the water/ethanol mutual diffusion coefficient Because the dynamics is slower in the nonideal systems, it takes somewhat longer times before these groups become qualitatively distinct. For the group of systems with the lowest value of *Đ*_12_ (colored with a blue shade in Figure 6), the supersaturation in the solvent side increases much more slowly, as compared to that for the group of systems with the highest value of *Đ*_12_ (colored with a red shade in Figure 6). In addition, the peak of the local supersaturation in the blue group gradually increases in height and slowly moves into the antisolvent mixture, eventually reaching the right side of the channel at a value slightly over the final, fully mixed supersaturation value. For the red group, the peak remains relatively stationary, even slightly moving into the solvent, as the supersaturation curve flattens.

**Figure 7:**
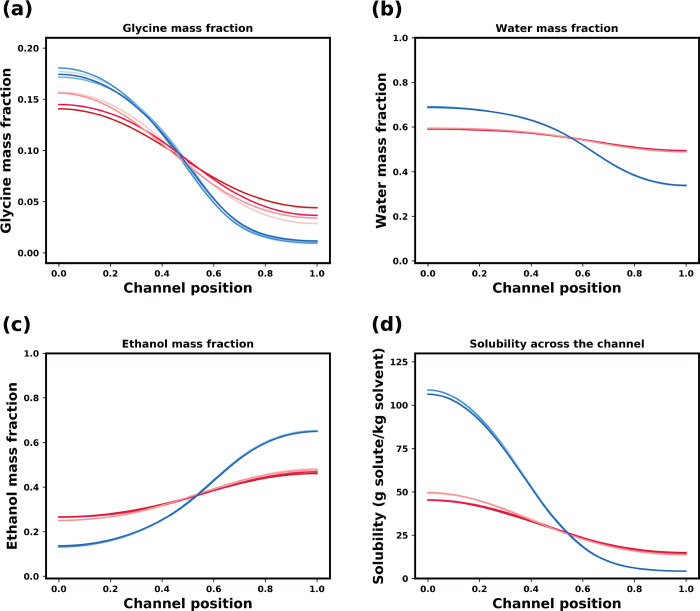
Mass fraction profiles in nonideal systems at 300 s for (a) glycine, (b) water, and (c) ethanol. (d) Solubility of glycine in the solvent mixture. The initial supersaturation in the solvent is 0.89. The initial antisolvent composition was 80% ethanol and 20 wt % water. The color scheme is the same as in Figure 6a, in which blue is slow antisolvent/solution diffusivity, and red is fast.

To gain more insight into the cause of this behavior, the mass fraction profiles of each species across the channel at 300 s are shown in Figure 7. The concentration profiles for each species are very similar within each group. Figure 7a shows that the glycine in the red group (fast solvent/antisolvent diffusivity) has diffused relatively quickly into the antisolvent, in comparison to the blue group (slow solvent/antisolvent diffusivity). This indicates the intermixing of water/ethanol helps to facilitate the diffusion of glycine. The movement of glycine into the antisolvent incurs a large chemical potential penalty (i.e., the local chemical potential would increase as glycine diffuses into ethanol), and, consequently, glycine retreats further to the solvent solution to avoid contact with ethanol. As the water content in the antisolvent increases, this penalty is significantly reduced, and glycine starts to diffuse into the antisolvent mixture. Another consideration is the effect of diffusing water “dragging” the glycine along with it as it intermixes with ethanol. Both of these phenomena lead to faster diffusion of glycine into the antisolvent in the red group in relation to the blue group.

From the relative amounts of glycine in the antisolvent, one would expect the red group to have a supersaturation peak at the channel wall on the antisolvent side of the system. However, if we consider the local composition, we can see why this happens for the blue group. The intermixing of the solvents results in low local solubility in the antisolvent side of channel, and hence with even low amounts of glycine, supersaturation is generated. Additionally, in the blue group, the slow diffusion of ethanol into the solution only causes a modest rise in supersaturation. The location of the supersaturation peak is, therefore, dependent on both glycine composition and local solvent composition and thus solubility.

This suggests that regardless of precise values of mutual diffusion coefficients, it is very unlikely that significant supersaturation overshoots would occur due to diffusive mixing in antisolvent crystallization processes. Therefore, predictions based on ideal solution models and Fickian diffusion and corresponding intuitions are physically incorrect.

### 5.3 Peak Supersaturation Trajectories in Ternary Phase Diagram

**Figure 8:**
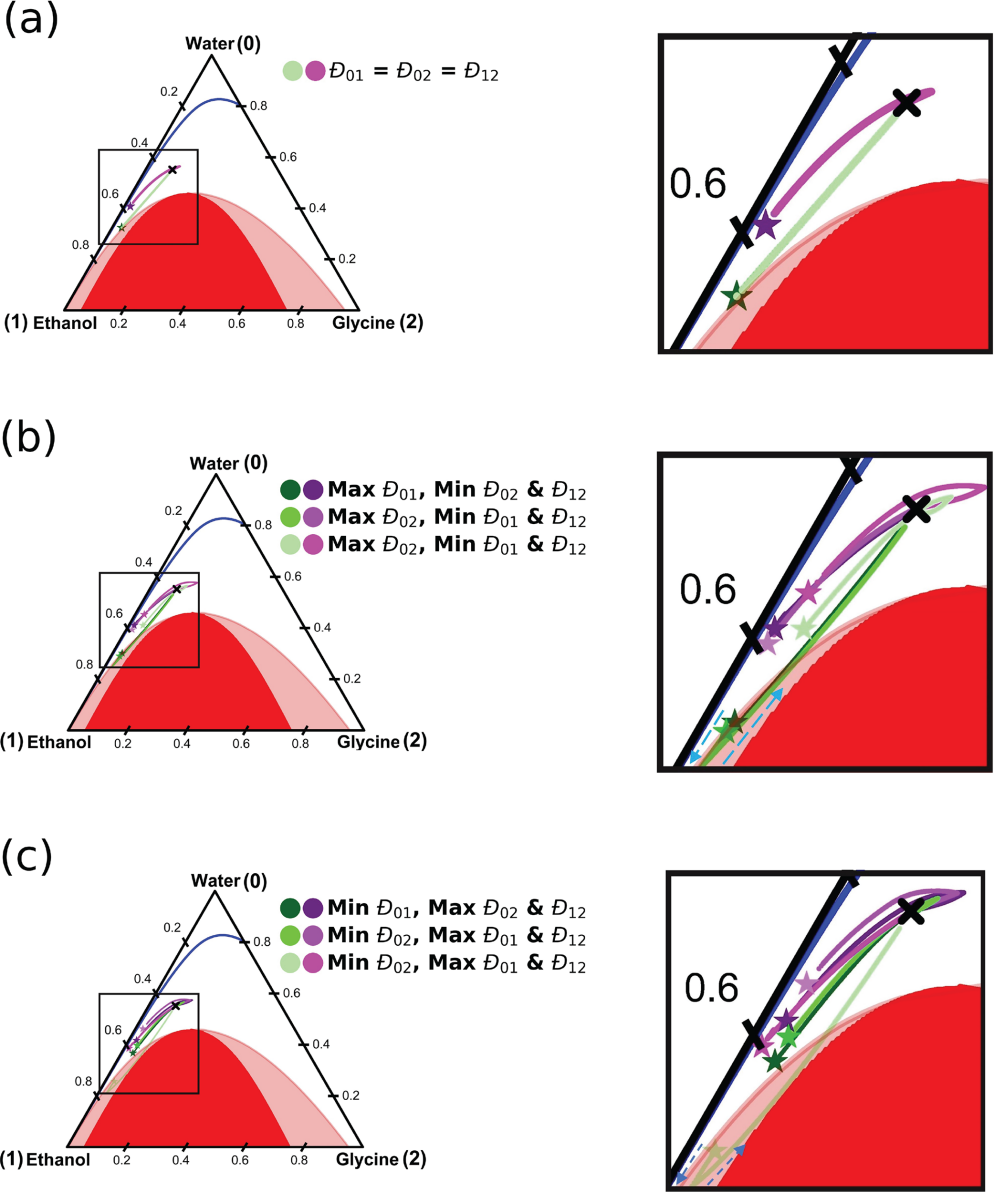
Ternary phase diagram showing the trajectories of the peak supersaturation compositions within the channel with respect to time. The stars indicate initial location of the supersaturation peak, while the black cross is the final fully mixed composition of the system. The blue dashed lines in panels b and c highlight that the trajectories initially move toward the ethanol corner, before changing direction. The sets of diffusion coefficients used relate to those in Figures 5 and 6. The initial supersaturation in the solvent is 0.89 for the nonideal systems, and 0.85 in the ideal systems. The initial antisolvent composition was 80 wt % ethanol and 20 wt % water. Green and purple trajectories refer to ideal and nonideal models, respectively.

Crystal nucleation is most likely to occur in the system at the peak of the local supersaturation. In Figure 8, the compositional trajectory of the supersaturation peak with respect to time is plotted on a mass based ternary phase diagram. Each point represents a time step of 1 s. Details of the calculations are provided in the Supporting Information. The trajectories for the eight sets of diffusivity values are divided into three groups:

(A) **All diffusion coefficients are the same:** This includes the two extreme combinations in which all three coefficients were set to the maximum and minimum values. The case where the diffusion coefficients are equal to 10^–9^ m^2^ s^–1^ (the “reference point”) was added to this group. This relates to Figure 8a.
(B) **One diffusion coefficient is significantly lower:** This is the case in which the diffusivity of one of the components was much lower than the other two in the mixture. These are the combinations in which one of the diffusion coefficients is chosen at the minimum of the range, while the other two are selected to be the maximum. This relates to Figure 8b.
(C) **One diffusion coefficient is significantly higher:** Similar to B, but with one maximum and two minimum values being selected. This relates to Figure 8c.

In group A (shown in Figure 8a), no significant differences can be seen in the predicted trajectories among the three cases for both ideal (shown in green) and nonideal (shown in purple) model. This is expected, as all three diffusion coefficients have the same value, so the relative fluxes and the compositional trajectories are identical, while only time scale affected (the differences in diffusive mixing times between ideal and nonideal solutions are highlighted in Figure 4). The ideal solution model predicts much higher concentrations of glycine and a higher concentration of ethanol at the peak supersaturation mixture. This again highlights that supersaturation in the ideal solution is driven by the glycine moving into the antisolvent in which solubility is very low. The trajectory for the nonideal system initially bypasses the final solvent mixture composition due to the movement of glycine into the aqueous solution, as it avoids the antisolvent. The peak supersaturation is generated by an increased glycine concentration in the solution. As the water and ethanol intermix, glycine diffuses into the antisolvent mixture, and the final composition is reached.

Figure 8b and c shows a range of trajectories for both the ideal and nonideal model. First, the nonideal model will be considered. Here, two behaviors are observed: one similar to the group A and the opposite case, in which no initial bypass of the final mixture composition is present. It is found that all combinations with the maximum diffusion coefficient value for water/ethanol lead to this bypass in the trajectory. This corresponds to the red group in Figure 7. The opposite is found to be true for combinations with the minimum coefficient for water/ethanol (blue group). This is explained by considering the cause of the peak supersaturation. For the red group, the fast mixing of the solvent and antisolvent lowers the local solubility of the solution, where the glycine concentration remains high. In addition, until the antisolvent and solvent are sufficiently mixed, only small amounts of glycine move into the antisolvent. For the blue group, water and ethanol mix slowly with respect glycine. Supersaturation is generated by glycine being present in regions with low local solubility. As water and ethanol mix, the final composition is approached. It is clear from the trajectories and the supersaturation profiles Figure 7 that the solvent/antisolvent diffusion coefficient plays a significant role in determining mixing profiles.

In the ideal systems, two behaviors were observed to occur. This was also based on the mutual diffusion coefficient of water/ethanol. For combinations in which water/ethanol diffusion was minimized, the trajectory initially moves toward the ethanol corner of phase diagram. This reflects that the supersaturation is caused by glycine moving into the low solubility antisolvent. As glycine reaches the channel wall, its mass fraction increases. Concomitantly, the mass fraction of water at the peak supersaturation increases as water/ethanol mix. From here, the trajectory moves to the final composition. In the opposite case, the trajectory bypasses the final composition in a similar manner to the nonideal case. The water/ethanol mix relatively quickly, and the peak supersaturation in the first instance is caused by antisolvent lowering the local solubility of the glycine solution. This is seen in the trajectory, where the starting composition is more concentrated in water. As glycine catches up with ethanol, the trajectory approaches the final composition.

In summary, the modeling approach developed here describes compositional and supersaturation profiles due to diffusive mixing in nonideal ternary mixtures during the induction time preceding any crystal formation. Although crystallization itself is not considered here, this model providing valuable insights into diffusive mixing in antisolvent crystallization within realistic nonideal solutions.

### 5.4 Liquid–Liquid Phase Separation

While the peak supersaturation trajectories in the ideal systems (shown in green in Figure 8) stay relatively close to the line connecting the initial compositions of the solution and antisolvent mixtures, in nonideal systems (shown in purple in Figure 8) they can explore a much wider range of ternary mixture compositions. Interestingly for nonideal systems, the composition profile can potentially enter the liquid–liquid phase coexistence region (which is denoted by the red shaded body in the ternary phase diagram in Figure 2), even though the final mixture composition may be well outside the liquid–liquid phase coexistence region. In particular, if the composition trajectory enters the spinodal region, it would directly lead to localized instantaneous liquid–liquid phase separation (LLPS) in the mixture.

Typically each of the coexisting liquid phases would have very different compositions,^35^ and because crystallization is strongly dependent on the local mixture compositions, LLPS can significantly influence crystal nucleation and growth and affect the outcomes of antisolvent crystallization. The presence of LLPS, even if only local or intermittent, can have a profound effect on the particular crystal polymorph that forms and the resulting particulate attributes in an antisolvent crystallization process. For example, in the continuous antisolvent crystallization of lovastatin, McGinty et al.^36^ found that the size and aspect ratio of the needle-like crystals formed could be controlled by altering the process conditions. Spinodal decomposition was suggested as a possible explanation for the change in aspect ratio, where under certain conditions nucleation and growth occurs inside “oiled out” droplets at local compositions different from the overall mixture composition,^37,38^ resulting in different crystal morphologies.

**Figure 9:**
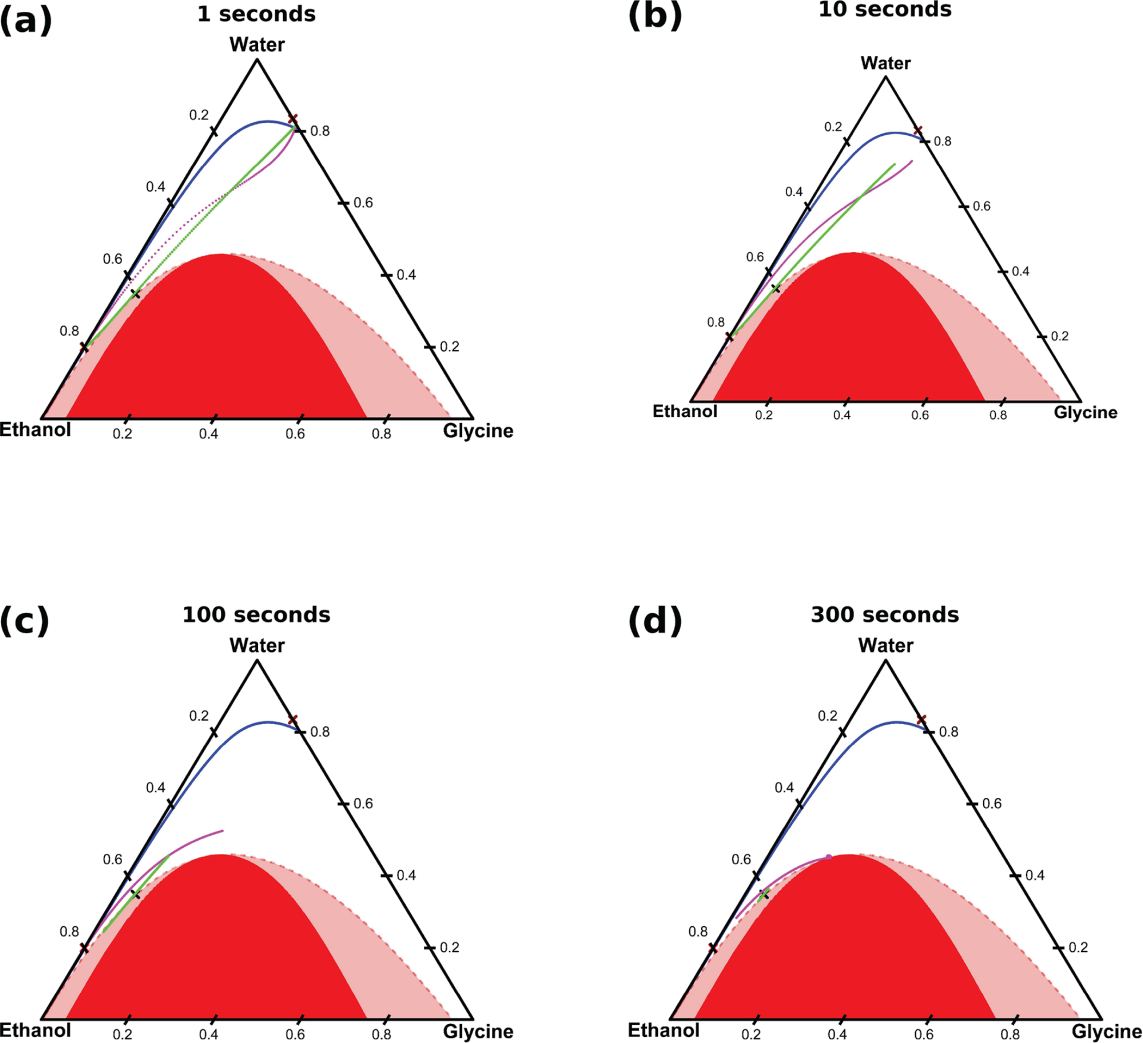
Example of predicted liquid–liquid phase separation during diffusive mixing. The run is stopped when the spinodal curve is encountered, as the model is not capable of simulating the actual demixing process. The antisolvent consisted of 80 wt % ethanol and 20 wt % water, the initial supersaturation was 0.89, and the channel was filled with an initial volume ratio of 7:3 antisolvent to solution. Each of the mutual diffusion coefficients is assumed to have a constant value of 10^–9^ m^2^ s^–1^. The composition profile across the channel for the nonideal case is shown in purple; the composition profile for the ideal case is depicted in green, and is shown for comparative purposes only, as liquid−liquid phase separation does not occur in ideal solutions. The red crosses denote the starting compositions of the antisolvent and solutions.

Consequently, it is crucial to understand when LLPS could potentially occur. When the overall liquid mixture composition is in the spinodal (or binodal) region during antisolvent crystallization, LLPS can occur and is usually observed as macroscopic “oiling out”; however, LLPS can also be present, but less apparent, when spinodal decomposition or rapid nucleation of the second liquid phase turns the system turbid, which is then followed by subsequent solid phase nucleation, before macroscopic oiling out can be observed. To investigate the scenario where the overall mixture composition is outside of the liquid–liquid phase coexistence region, but there is localized LLPS during diffusive mixing, a set of conditions was chosen where the peak supersaturation position reaches the spinodal curve. The evolution of the composition profile across the channel with time is shown in Figure 9.

In this scenario, localized spinodal decomposition is predicted in the course of diffusing mixing. It is worth noting that this is not due to supersaturation with respect to the solid phase, as this remains similar to the final fully mixed value, although local solvent mixture compositions can be rather different from the final fully mixed conditions. Instead, this is due to the solution becoming locally thermodynamically unstable with respect to the liquid–liquid separation, so that localized LLPS would be instant due to spinodal decomposition.

The nonideal diffusive mixing model developed here can provide quantitative information on conditions that are likely to cause the local LLPS in nonideal ternary solutions. This will provide novel insights into LLPS phenomena and their role in antisolvent crystallization processes.

## 6 Conclusions

Antisolvent crystallization systems are highly nonideal, and assuming ideality in modeling of diffusive mixing fails to provide a qualitatively accurate representation of local composition and supersaturation profiles. Diffusive mixing in ideal solutions relies in the Fickian framework where the driving force for diffusion is based on concentration gradients rather than chemical potential gradients, leading to unphysical predictions of local, large overshoots in the local supersaturation profile with respect to that in the final, fully mixed system. A detailed study on the diffusive mixing of an antisolvent system consisting of glycine/water/ethanol was performed for both ideal and nonideal solutions. Qualitative differences were observed between models with the large supersaturation overshoots predicted in the ideal model, but not by the nonideal model. This is caused by solute initially diffusing away from the antisolvent, against the concentration gradient, as dictated by the chemical potential gradient, based on nonideal solution thermodynamics. It was also found that, for certain conditions, localized liquid–liquid spinodal demixing can be expected during diffusive mixing, even when the final mixture composition would suggest otherwise. While diffusive mixing is unlikely to lead to significant sensitivity of nucleation to mixing conditions, intermittent spinodal demixing driven by diffusive mixing may provide a novel explanation for differences of nucleation behaviors among various antisolvents.

## Supporting Information Available

Model system description, Fickian diffusion coefficient, time-step checks, ideal parametric study, and nonideal parametric study (PDF)
