# Supplementary material for: Modeling Diffusive Mixing in Antisolvent Crystallization
Source: Cryst Growth Des. 2022 Mar 14;22(4):2192–207. doi: 10.1021/acs.cgd.1c01269 (PMC9007412; doi:10.1021/acs.cgd.1c01269)
Supplement: Supplementary file 1 — cg1c01269_si_002.pdf [file cg1c01269_si_002.pdf]

Modeling Diffusive Mixing in AntiSolvent Crystallisation -Supporting Information

Russell Miller,[*,†,‡] Jan Sefcik,[†,‡] and Leo Lue[†]

† *Department of Chemical and Process Engineering, University of Strathclyde, James Weir Building, 75 Montrose Street, Glasgow G1 1XJ, UK*

‡ *EPSRC Continuous Manufacturing & Advanced Crystallisation (CMAC) Future Manufacturing Research Hub, University of Strathclyde, Glasgow G1 1RD, UK*

E-mail: russell.miller@strath.ac.uk

Methodology

Model system

To model the diffusive mixing in anti-solvent crystallisation, a ternary component system consisting of water, ethanol and glycine is considered. In this system, glycine is the solute, water is the solvent and a mixture of ethanol/water was used as the anti-solvent. The system was modelled as a static channel, with one part filled with aqueous glycine solution, and the other part the anti-solvent/solvent mixture. Figure 1 describes an example of the initial volume fraction profile of the channel. Note the volume fraction refers to $\phi_i = \frac{V_i \cdot x_i}{\sum_j^N V_i \cdot x_i}$, where V_i refers to the pure component volume of species i, x_i is the mol fraction and superscript N is the number of species in the mixtures.

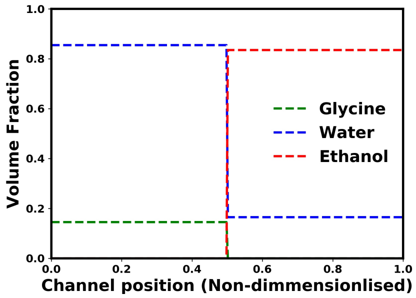

Figure 1: Example of the initial volume fraction profile within the simulated channel. This was for a 50:50 initial ratio of solution to antisolvent within the channel. Antisolvent composition was 83.5% ethanol and 16.5% water.

Diffusion Coefficients

The Fickian mutual diffusion coefficients for Glycine-water[1–6] and ethanol-water[7–11] mixtures, are shown in figure2.

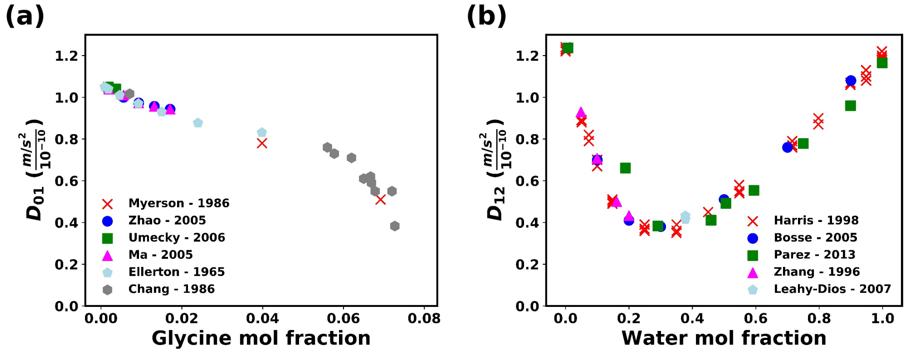

Figure 2: Literature values of binary Fickian diffusion coefficients for (a): Water/glycine (D_{01}) and (b): Water/Ethanol (D_{12}) Both are in molar reference frame.

Time-Step checks

The time-steps used in the simulations were 0.001s and 0.1s. To check this did not effect the accuracy of the results, simulations were performed for time-steps of 0.001s and compared to 0.1s. Figure 3 shows the volume fraction profiles at short times.

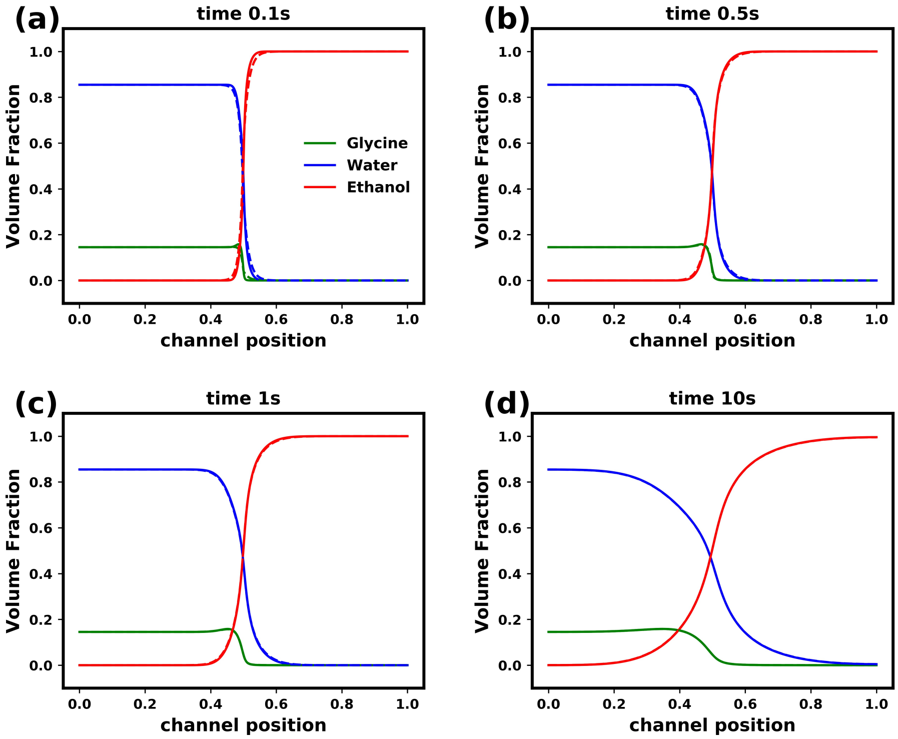

Figure 3: Comparison of volume fraction (ϕ) profiles for time steps 0.1 and 0.001s. Pure ethanol was used as the antisolvent, the solution to antisolvent ratio was 1:1 within the channel and the initial supersaturation was 0.9. Dotted lines indicate 0.1 and solid lines represent 0.001s. The non-ideal solution model was used.

Figure 3a shows slight differences in the volume fractions around the interface. As time progresses, the profiles smooth out, and the volume fractions become independent of time-step used. The corresponding supersaturation profiles were plotted, shown in figure 4.

At 0.1s (figure 4a) shows a large difference between the profiles, with the time-step of 0.1 predicting a large overshoot in local supersaturation. After 0.5s, this overshoot is no longer present. Although the volume fraction profiles vary marginally, the calculated supersaturation profiles are sensitive to the composition profiles, reflected by contrasting

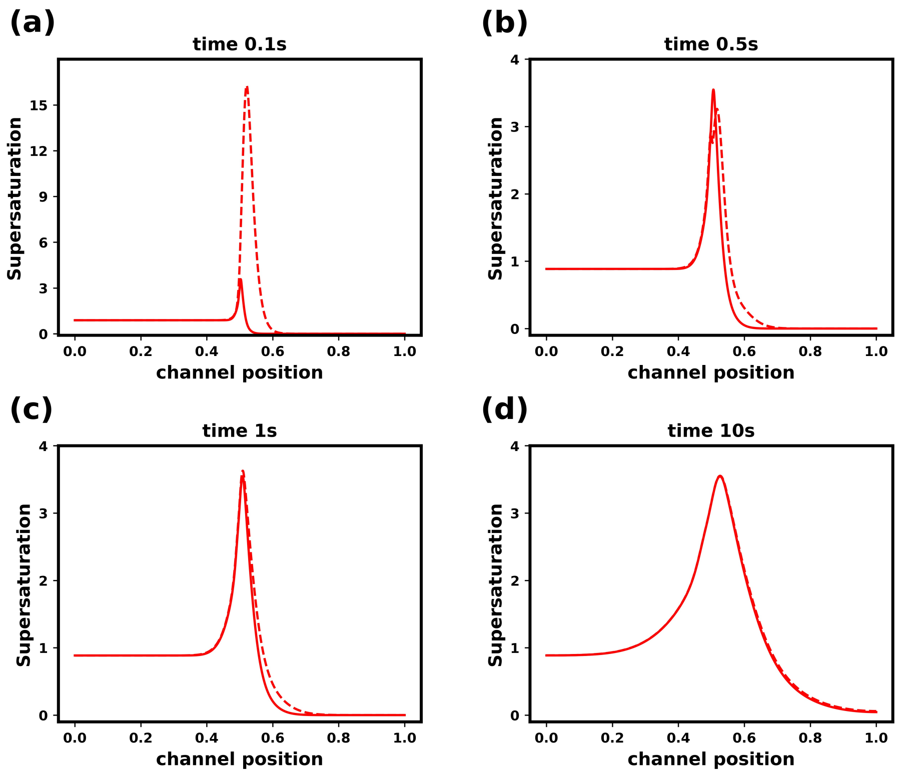

Figure 4: Comparison of supersaturation profiles for time steps 0.1 and 0.001s. Pure ethanol was used as the antisolvent, the solution to antisolvent ratio was 1:1 within the channel and the initial supersaturation was 0.9. Supersaturation is defined as $a_{glycine}/a_{glycine,sat}$. Dashed lines indicate 0.1 and solid lines represent 0.001s. The purple line is the final 'fully mixed' supersaturation.

figures 3 & 4. For times greater than 1s there is no qualitative differences in the behaviour of the system.

To capture earlier behaviour accurately, the time step used was 0.001s. At 20s this was reduced to 0.1s as no differences in mass fraction profiles were present.

Results

Ideal parametric study

To further develop understanding of diffusive mixing in antisolvent crystallisation, a parametric study was performed over three key process parameter. These were antisolvent compo-

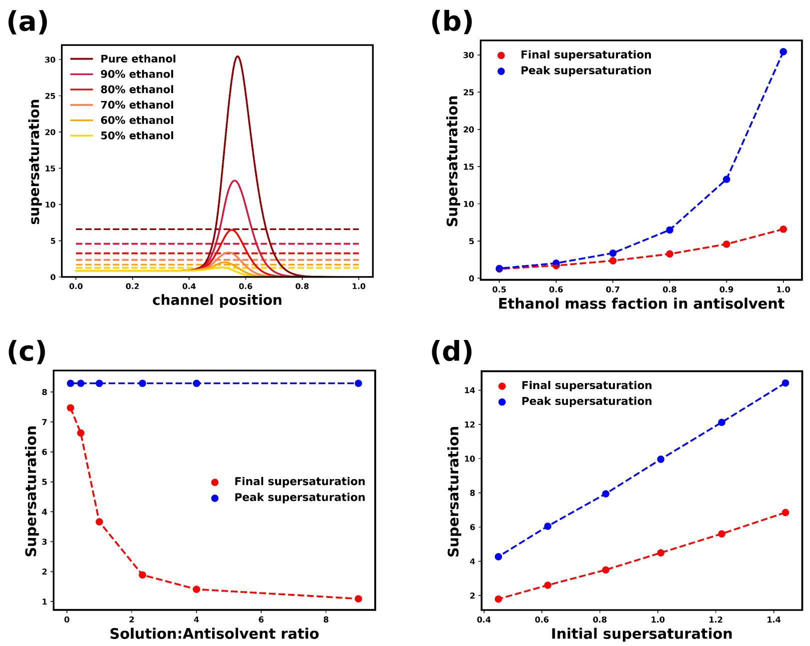

Figure 5: Summary of effect of key process parameters. (a) Effect of antisolvent composition after 1s after mixing. Dotted lines indicate 'final fully mixed' supersaturation. (b) - (d) show the highest supersaturation that is experienced during the mixing process (blue), in comparison to the final value (red). This assumes no crystallisation. Dotted lines are aesthetic only, and do not represent a predictive model. (b): initial antisolvent composition, (c): Solution to antisolvent ratio & (d) initial supersaturation. All supersaturations are expressed in the ratio of local activity to saturation activity.

sition, ratio of solution to antisolvent and the initial supersaturation. Figure 5 summarises the results of this study. To quantify the comparisons across the parameter values, the maximum supersaturation attained during mixing is plotted along with the fully mixed supersaturation. This gives insight into how nucleation conditions are impacted by the relative parameter, along with the relative size of the 'overshoot' in supersaturation. It also highlights that mixing should be carefully considered in the design of crystallisation processes, as this influences crystallisation outcomes. The movement of the peak supersaturation into the antisolvent is present across all parameters, hence this was not considered in this analysis. As one parameter is varied, the others are held constant at 'centre point' values. Table 1 summarises this.

Figure 5a shows how the peak supersaturation is effected by initial antisolvent compo-

Table 1: Key process 'centre point' values. This indicates the values of key process parameters used when not being varied. Antisolvent is mixture of ethanol and water.

Parameter	Value
Ethanol mass fraction in antisolvent	0.80
Solution:Antisolvent ratio	1:1
Initial Supersaturation	0.85

sition. A clear trend is observed, with antisolvent with higher ethanol content relating to greater supersaturations. The trend in the ratio of peak to final supersaturation is more apparent in figure 5b. Larger overshoots are present in the pure antisolvent, which becomes increasingly lower as the water content increases. From intuition this is expected. In the ideal model, supersaturation reduces to the ratio of mol fraction of glycine and the saturated value, i.e. solubility. Glycine diffuses down its composition gradient and the overshoot in supersaturation will be dictated by the local solubility of the antisolvent. Solubility is extremely low in the pure antisolvent and thus large overshoots are predicted. In many cases, these overshoots are undesirable, for example effective secondary nucleation processes require control over supersaturation. High levels of supersaturation can lead to primary nucleation, resulting in unwanted crystal properties. For antisolvents that have higher water content, the peak supersaturation decreases with respect to the final value. This may help in controlling nucleation. The driving force for crystallisation is supersaturation, so an antisolvent composition should be selected to balance control and to achieve a suitable nucleation rate.

Figure 5c is the effect of varying the solution:antisolvent ratio. The peak supersaturation at early times was found to be independent of this ratio. This agrees with the study by Thorson[12] as discussed in main paper.. In terms of diffusion, this can be explained by considering short time behaviour at the interface. Immediately after the onset of mixing glycine diffuses into the antisolvent and an overshoot in supersaturation is observed. At distances away from the interface, the glycine does not start to diffuse until there is a driving force present. i.e. the compositional gradient will be flat within regions away from

the interface at short times. As diffusion proceeds at the interface a compositional gradient is generated, as glycine moves into the antisolvent. The same reasoning can be applied to the diffusion of water and ethanol. At long times, the ratio of solution:antisolvent impacts the supersaturation profile, which can be seen in the final supersaturation. A straight forward relationship is seen, with more antisolvent leading to higher final supersaturations. This is simply due to the lower solubility of the solvent mixture. One limitation of this study is the exclusion of nucleation. Nucleation would act to lower the supersaturation, and if it is sufficiently high at the interface, then long term predicted profiles could fail to represent the physical system. Quantitative behaviour however, would still be expected to remain reasonably accurate.

The last parameter investigated was the initial supersaturation, which is essentially equivalent to the initial mass fraction of glycine in the solution. The trends in both peak and final supersaturation are once more what would be expected from intuition. That is, increased glycine in the initial solution results in an increased supersaturation in the fully mixed system. The peak supersaturation follows the same trend. This is caused by the increased driving force for the diffusion of glycine. This means more glycine would be present in the antisolvent thus, greater degree of supersaturation.

Non-ideal parametric study

The effects of key processes parameters were studied for mixing in ideal solutions, however the previous sections have shown that the ideal model does not represent the physical process. To correct this, the activity gradient was considered as the driving force. Figure 6 details the effect of these parameters.

Antisolvent composition (6b) indicates that the peak supersaturation experienced is only slightly greater than the final value. Additionally the 'overshoot' is no longer present initially after mixing as is with the ideal case, but occurs at the antisolvent channel wall towards the end of mixing. This suggests that large overshoots in supersaturation as expected through

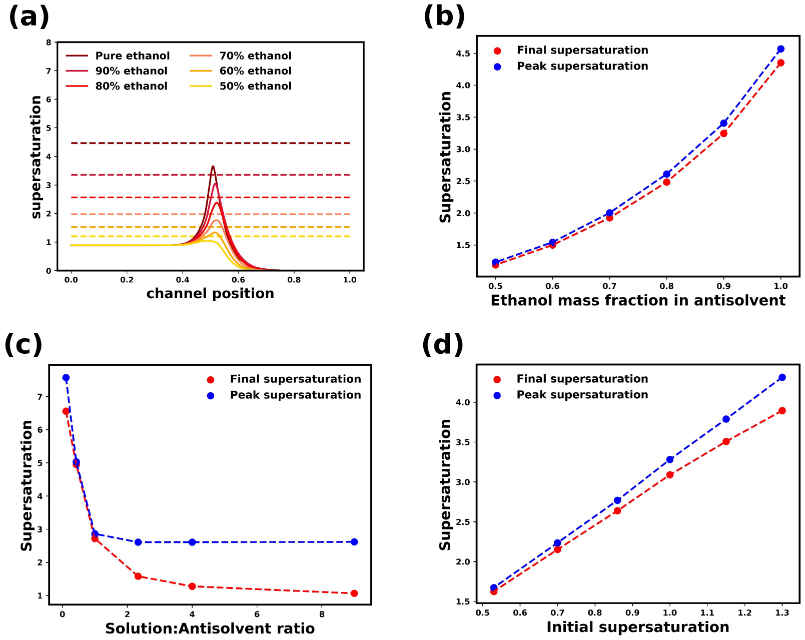

Figure 6: Summary of effect of key process parameters. (a) Effect of antisolvent composition after 1s after mixing. Dotted lines indicate 'final fully mixed' supersaturation. (b) - (d) show the highest supersaturation that is experienced during the mixing process (blue), in comparison to the final value (red). This assumes no crystallisation. Dotted lines are aesthetic only, and do not represent a predictive model. (b): initial antisolvent composition, (c): Solution to antisolvent ratio & (d) initial supersaturation. All supersaturations are expressed in the ratio of local activity to saturation activity.

intuition do not exist. However, figure 6a highlights that the initial peak supersaturation increases with increasing ethanol wt % in the antisolvent, similarly to the ideal case, as does the value of the maximum supersaturation attained during mixing.

The ratio of solution:antisolvent again produced results similar to the ideal model. For ratios of 1:1 or greater the peak supersaturation is approximately constant. These ratios the initial peak is located at the interface between the solution and antisolvent, and was found to be independent of solution:antisolvent ratio. At small time scales, mixing is only experienced at the interface and this is expected. Low amounts of relative antisolvent lead to the final supersaturation being relatively low, and the initial peak is found to be the highest in magnitude. For ratios less than one, the peak value is realised due to high antisolvent composition in the final mixture. For the ratio of 1:3 (solution:antisolvent) the largest

overshoot occurs as the antisolvent mixes into the solution. Glycine moves relatively slow in comparison to the water/ethanol intermixing and hence supersaturation is generated as ethanol lowers local solubility at the solution wall.

Quantitative trends were unchanged from the ideal and non-ideal models with higher initial supersaturation (initial glycine mass fraction) producing higher supersaturations. The key difference between models is once more the magnitude of the overshoots. In the non-ideal model these are small, and increase with higher supersaturations as more glycine is present. Spatiotemporal profiles are shown in the

When comparing trends of key process parameters for ideal and non-ideal models we find they are similar. However a key difference is observed when considering the magnitudes of peak supersaturations. The non-ideal model predicts more modest peaks. The composition of the peak varies between models and therefore significantly different nucleation conditions are modelled. The ideal model therefore fails to predict real-life physical process, even if the effects of key process parameters make sense intuitively.

References

(1) Myerson, A. S.; Chang, Y. C. Diffusional separation in ternary systems. *AIChE J.* **1986**, *32*, 1747–1749.

(2) Zhao, C. W.; Li, J. D.; Ma, P. S.; Xia, S. Q. Measurement of liquid diffusion coefficients of aqueous solutions of glycine, L-alanine, L-valine and L-isoleucine by holographic interferometry. *Chin. J. Chem. Eng.* **2005**, *13*, 285–290.

(3) Umecky, T.; Kuga, T.; Funazukuri, T. Infinite dilution binary diffusion coefficients of several α-amino acids in water over a temperature range from (293.2 to 333.2) K with the Taylor dispersion technique. *J. Chem. Eng. Data* **2006**, *51*, 1705–1710.

(4) Ma, Y.; Zhu, C.; Ma, P.; Yu, K. T. Studies on the diffusion coefficients of amino acids in aqueous solutions. *J. Chem. Eng. Data* **2005**, *50*, 1192–1196.

(5) Ellerton, H. D.; Reinfelds, G.; Mulcahy, D. E.; Dunlop, P. J. The mutual frictional coefficients of several amino acids in aqueous solution at 25C. *J. Phys. Chem.* **1964**, *68*, 403–408.

(6) Chang, Y. C.; Myerson, A. S. Cluster diffusion in metastable solutions. *AIChE J.* **1987**, *33*, 697–699.

(7) Harris, K. R.; Newitt, P. J.; Derlacki, Z. J. Alcohol tracer diffusion, density, NMR and FTIR studies of aqueous ethanol and 2,2,2-trifluoroethanol solutions at 25°C. *J. Chem. Soc. Faraday Trans.* **1998**, *94*, 1963–1970.

(8) Bosse, D.; Bart, H.-J. Measurement of Diffusion Coefficients in Thermodynamically Nonideal Systems. *J. Chem. Eng. Data* **2005**, *50*, 1525–1528.

(9) Pařez, S.; Guevara-Carrion, G.; Hasse, H.; Vrabec, J. Mutual diffusion in the ternary mixture of water + methanol + ethanol and its binary subsystems. *Phys. Chem. Chem. Phys.* **2013**, *15*, 3985–4001.

(10) Zhang, K. J.; Briggs, M. E.; Gammon, R. W.; Sengers, J. V. Optical measurement of the Soret coefficient and the diffusion coefficient of liquid mixtures. *J. Chem. Phys.* **1996**, *104*, 6881–6892.

(11) Leahy-Dios, A.; Firoozabadi, A. Molecular and thermal diffusion coefficients of alkane-alkane and alkane-aromatic binary mixtures: Effect of shape and size of molecules. *J. Phys. Chem* **2007**, *111*, 191–198.

(12) Thorson, M. R.; Goyal, S.; Gong, Y.; Zhang, G. G. Z.; Kenis, P. J. A. Microfluidic approach to polymorph screening through antisolvent crystallization. *CrystEngComm* **2012**, *14*, 2404.
